# The Antitumour Activity of a Curcumin and Piperine Loaded iRGD-Modified Liposome: In Vitro and In Vivo Evaluation

**DOI:** 10.3390/molecules28186532

**Published:** 2023-09-09

**Authors:** Yingzheng Wang, Xunhua Huang, Hanzhi Chen, Qianyuan Wu, Qianqian Zhao, Dezhuang Fu, Qinghua Liu, Yinghao Wang

**Affiliations:** College of Pharmacy, Fujian University of Traditional Chinese Medicine, Fuzhou 350122, China; wangyingzheng@live.com (Y.W.); hxh271555684@126.com (X.H.); yxhm1062604385@126.com (H.C.); wqy657889931@126.com (Q.W.); zqq357115525@126.com (Q.Z.); f1021827058@126.com (D.F.)

**Keywords:** iRGD peptide-modified liposome, curcumin, piperine, synergistic antitumour activity, lung cancer background

## Abstract

Lung cancer is one of the most common cancers around the world, with a high mortality rate. Despite substantial advancements in diagnoses and therapies, the outlook and survival of patients with lung cancer remains dismal due to drug tolerance and malignant reactions. New interventional treatments urgently need to be explored if natural compounds are to be used to reduce toxicity and adverse effects to meet the needs of lung cancer clinical treatment. An internalizing arginine-glycine-aspartic acid (iRGD) modified by a tumour-piercing peptide liposome (iRGD-LP-CUR-PIP) was developed via co-delivery of curcumin (CUR) and piperine (PIP). Its antitumour efficacy was evaluated and validated via in vivo and in vitro experiments. iRGD-LP-CUR-PIP enhanced tumour targeting and cellular internalisation effectively. In vitro, iRGD-LP-CUR-PIP exhibited enhanced cellular uptake, suppression of tumour cell multiplication and invasion and energy-independent cellular uptake. In vivo, iRGD-LP-CUR-PIP showed high antitumour efficacy, mainly in terms of significant tumour volume reduction and increased weight and spleen index. Data showed that iRGD peptide has active tumour targeting and it significantly improves the penetration and cellular internalisation of tumours in the liposomal system. The use of CUR in combination with PIP can exert synergistic antitumour activity. This study provides a targeted therapeutic system based on natural components to improve antitumour efficacy in lung cancer.

## 1. Introduction

Lung cancer is a highly malignant tumour and one of the most common types of cancer worldwide. In 2020, about 2.207 million new lung cancer cases and about 1.796 million deaths from lung cancer were reported [1]. The current treatment for lung cancer mainly includes surgery to remove the lesion, chemotherapy, targeted therapies and immunotherapeutic agents. However, all these treatments have several side effects on patients with lung cancer [2,3,4]. Therefore, exploring new interventions to improve lung cancer treatment is an imperative task.

New therapeutic interventions have been considered in the treatment of lung cancer, such as using natural compounds that can reduce toxicity. Turmeric is the dried rootstock of the genus *Curcuma* in the family of ginger. Its use was first published in the newly revised *Materia Medica*. Curcumin (CUR) is a natural and effective ingredient extracted from curcuma, and has many pharmacology activities such as antineoplastic, anti-inflammatory, antioxidant liver protection and memory enhancement [5,6,7,8,9,10]. Remarkably, the anticancer property of CUR is one of the current research hotspots and has great promise in the field of antitumour [11]. New clinical data further confirm the significant potential of CUR in cancer treatment [12,13]. A study showed that CUR has significant anticancer effect on lung cancer and the ability to inhibit the proliferation, migration and infestation of lung cancer cells effectively [14,15]. However, CUR is chemically unstable and rapidly degrades at neutral and alkaline pH, resulting in poor water solubility, fast metabolism and systemic clearance, especially in the intestine and liver, resulting in poor absorption and limiting its clinical development and use [16,17]. In order to overcome these limitations by improving the solubility and bioavailability of CUR, natural enhancers and nanobiotechnology may be helpful [18,19], such as piperine (PIP) and liposome preparations [20]. PIP is derived from the dried near-ripe or ripe fruit of *Piper nigrum* L. of the Piperaceae family and it is the main chemically active substance. It can reduce the level of UDP-glucuronide in blood and inhibit glucuronidation. PIP is also a potent bioenhancer, which helps slow down the hepatic metabolism of CUR, thereby increasing its bioavailability [21]; exerts antitumour activity in multiple cancers; and enhances the anticancer activity of CUR [22,23].

Liposomes have a biofilm-like structure and can encapsulate water- and lipid-soluble drugs. As drug carriers, they are highly targetable and can effectively protect encapsulated drugs and control and slow the release of drugs. The tumour-homing peptide iRGD is a novel cyclic peptide composed of nine amino acids, including the Arg Gly Asp (RGD) motif, which is abundant in the tumour vascular system of αvβ3 and αvβ5 integrins, with high binding affinity [24,25]. iRGD can specifically bind to integrins and neurocorticolin-1 (NRP-1) receptors that are overexpressed in various tumours. The cleavage form of iRGD binds to NRP-1 and subsequently triggers NRP-1 dependent endocytosis, thereby enhancing tumour infiltration [26]. Surface coupling of iRGD to liposomes provides extra advantages of aggressive tumour targeting and tumour penetration, thereby improving therapeutic efficacy. In the present study, iRGD was chosen as the functional targeting substrate, CUR and PIP as the model drugs, and LP as the delivery system to produce CUR-PIP-loaded, iRGD-modified LP (iRGD-LP-CUR-PIP). A549 cells were selected as in vivo and ex vivo tumour cell models. iRGD-LP-CUR-PIP was further investigated for its antitumour activity.

## 2. Results and Discussion

### 2.1. Characterisation of iRGD-LP-CUR-PIP

**Figure 1:**
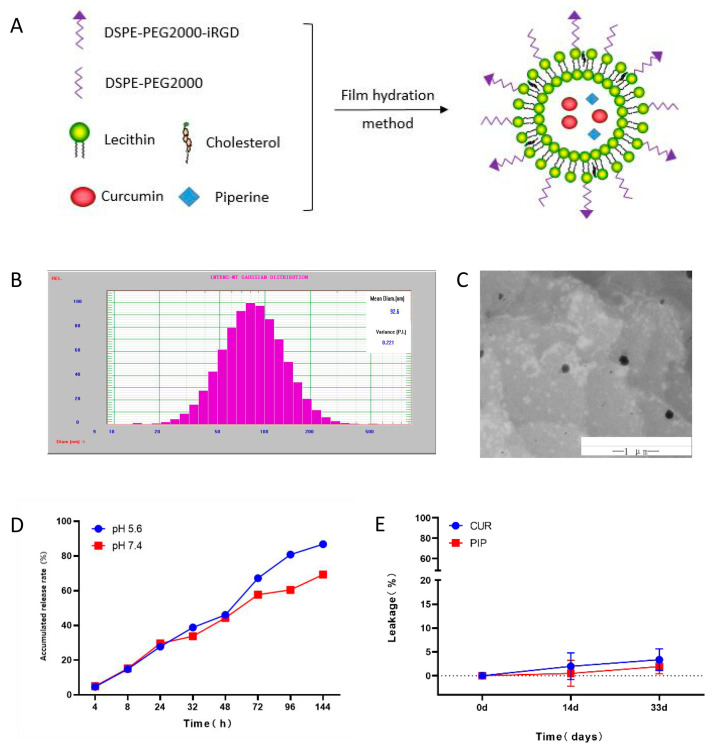
Characterisation of iRGD-LP-CUR-PIP. Notes: (**A**) Preparation of iRGD-LP-CUR-PIP. (**B**) Characteristic particle size or texture of iRGD-LP-CUR-PIP. (**C**) Transmission electron microscopic analysis showing spherical nanoparticles with a flat and smooth surface (scale bar = 200 nm). (**D**) iRGD-LP-CUR-PIP exhibiting sustained release in pH 5.6 and 7.4 PBS.©). (E) Leakage of CUR and PIP from iRGD-LP-CUR-PIP.

The preparation of iRGD-LP-CUR-PIP is shown in Figure 1A. The average grain diameter of iRGD-LP-CUR-PIP was about 93 nm ± 0.9 nm and the PDI was 0.2 ± 0.015. The zeta potential of iRGD-LP-CUR-PIP is shown in Table 1. The entrapment efficiency of iRGD-LP-CUR-PIP indicated that CUR and PIP were almost trapped within the liposomes. The typical particle size and distribution of iRGD-LS-CUR-PIP are shown in Figure 1B. The transmission electron microscopic images showed that the nanoparticles were spherical with a flat and smooth surface. They were evenly distributed without adhesion and agglomeration (Figure 1C). iRGD-LP-CUR-PIP sustained release was observed in PBS at pH levels 5.6 and 7.4 (Figure 1D).

The ©n-vitro leakages of CUR and PIP from iRGD-LP-CUR-PIP in PBS at 4 °C are shown in Figure 1E. The leakages of CUR and PIP at the 14-day timepoint were 2% and 0.52%, respectively. Even at 33 days, the leakages of CUR and PIP were only about 3.36% and 1.92%, respectively, demonstrating the stability of iRGD-LP-CUR-PIP. CUR is unstable under neutral and alkaline conditions, making it easily metabolised after entering the body, and its bioavailability is low, which greatly limits its clinical application. The preparation of PIP binding as liposomes significantly improves solubility and bioavailability. [27]. The results showed that the prepared iRGD- LP-CUR-PIP exhibited satisfactory particle size, distribution and in vitro leakage. The prepared iRGD-LP-CUR-PIP exhibited the slow-release properties of CUR and remained relatively stable for at least 33 days.

### 2.2. In Vitro Cellular Uptake

**Figure 2:**
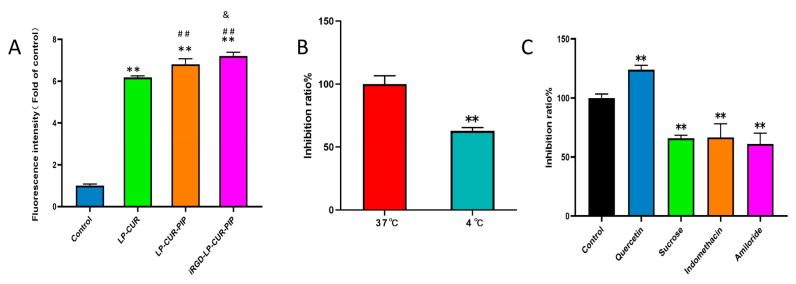
Cellular uptake characteristics of iRGD-LP-CUR-PIP. Notes: (**A**) Fluorescence intensity of A549 cells incubated with different CUR formulations (free CUR, LP-CUR, LP-CUR-PIP and iRGD-LP-CUR-PIP). ** *p* < 0.01 versus control, ^##^ *p* < 0.01 versus LP-CUR, ^&^ *p* < 0.05 versus LP-CUR-PIP. (**B**) Fluorescence intensity of iRGD-LP-CUR-PIP at 37 °C and 4 °C. ** *p* < 0.01 versus 37 °C. (**C**) Endocytosis pathway of iRGD-LP-CUR-PIP. ** *p* < 0.01 versus control.

As mentioned earlier, iRGD is a common tumour-active targeting peptide that can act directly on a tumour target to form an active targeting effect. The fluorescence intensity inside the cells was measured via stream cytometry to observe the uptake of different preparations. The fluorescence intensity of iRGD-LP-CUR-PIP was significantly higher than that of LP-CUR-PIP (Figure 2A). Further analysis showed that the fluorescence intensity at 37 °C was stronger than at 4 °C (Figure 2B), suggesting that the uptake behaviour was not physisorption nor passive diffusion but relied mainly on endocytosis. In other words, the cellular uptake was energy-dependent. Endocytosis inhibitors were separately preadded to investigate which endocytosis pathway liposomes entered the cells. The result showed that sucrose, indomethacin and amiloride reduced endocytosis instead of quercetin (Figure 2C), indicating that the liposomes mainly entered the cells in the form of clathrin- and caveolin-dependent endocytosis and micropinocytosis [28].

### 2.3. In Vitro Cytotoxicity

**Figure 3:**
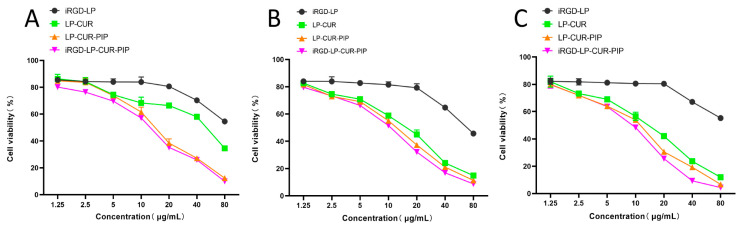
In vitro cytotoxicity of iRGD-LP-CUR-PIP. Notes: Viability of A549 cells after incubation with iRGD-LP, LP-CUR, LP-CUR-PIP and iRGD-LP-CUR-PIP for 24 (**A**), 48 (**B**) and 72 h (**C**).

The cells were treated with blank liposomes at 1.25–80 μg/mL concentrations for 24, 48 and 72 h. The blank liposomes at concentrations ranging from 1.25 μg/mL to 20 μg/mL had higher biological compatibility. Then, the cytotoxicity of the different formulations was determined via cell proliferation analysis after 24, 48 and 72 h of culture (Figure 3). The combination of CUR and PIP showed better cytotoxicity than CUR alone. The cytotoxicity of iRGD-LP-CUR-PIP was comparable to that of LP-CUR-PIP at 24 h and it strengthened after 48 and 72 h, indicating that iRGD-LP-CUR-PIP was time-dependent. The results showed that iRGD-LP-CUR-PIP exhibited significant cytotoxicity against a range of A549 cells in a time- and concentration-dependent manner.

### 2.4. Inhibition of In Vitro Invasion

**Figure 4:**
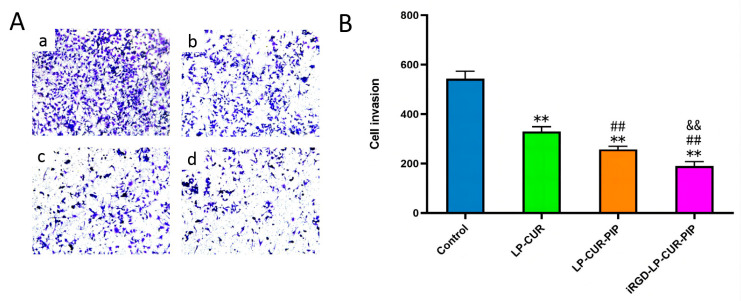
Inhibition of in vitro invasion of iRGD-LP-CUR-PIP. Notes: Invasion inhibition images (**A**), a, b, c and d correspond to control, LP-CUR, LP-CUR-PIP and iRGD-LP-CUR-PIP. (**B**) Analysis of A549 cells after incubation with PBS, LP-CUR, LP-CUR-PIP and iRGD-LP-CUR-PIP. ** *p* < 0.01 versus control, ^##^ *p* < 0.01 versus LP-CUR and ^&&^ *p* < 0.01 versus LP-CUR-PIP.

iRGD-LP-CUR-PIP significantly suppressed the invasion of A549 cells, and its inhibitory action was significantly better than that of LP-CUR and LP-CUR-PIP (Figure 4), which explains its potential ability to reduce tumour metastasis.

### 2.5. Biodistribution Characteristics of DiR-Loaded Liposomes

**Figure 5:**
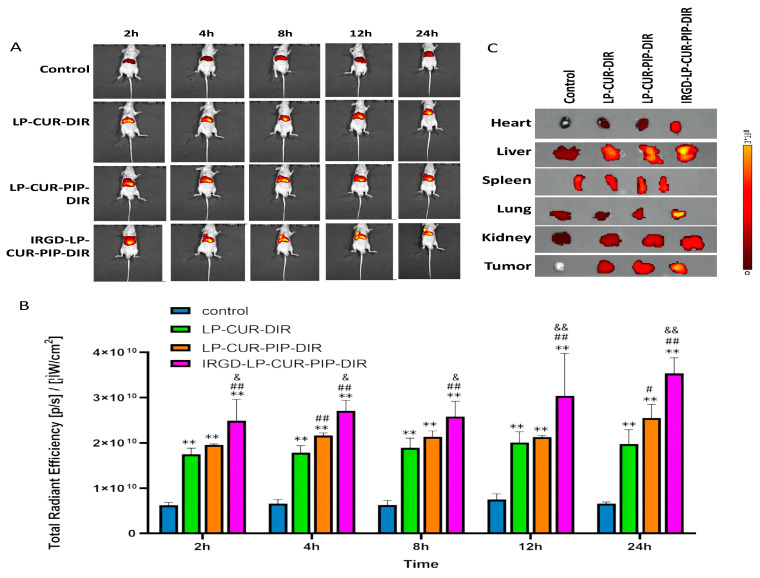
Fluorescence signal distribution analysis of iRGD-LP-CUR-PIP in ruffled mouse model. Notes: Mice were intravenously injected with free DiR, LP-CUR-DiR, LP-CUR-PIP-DiR and iRGD-LP-CUR-PIP via tail vein (after routine sterilisation). LP-CUR-PIP-DiR and iRGD-LP-CUR-PIP. Fluorescence images (**A**) and fluorescent-signal analysis (**B**) of mice treated with different Cy5 formulations in vivo at different timepoints (2, 4, 8, 12 and 24 h). ** *p* < 0.01 versus control, ^##^ *p* < 0.01 versus LP-CUR and ^&^ *p* < 0.05 and ^&&^ *p* < 0.01 versus LP-CUR-PIP. (**C**) Fluorescence images of each group of isolated organs and tumour tissues after 24 h following injection of different CUR formulations via tail vein.

The fluorescent DiR distribution and tumour accumulation in A549 tumour-bearing nude mice are shown in Figure 5A,B. The fluorescence distribution in the LP-CUR-DiR, LP-CUR-PIP-DiR and iRGD-LP-CUR-PIP-DiR groups was observed within 2 h compared with that in the control group. The iRGD-LP-CUR-PIP-DiR group showed fluorescence attachment at the tumour site at 4 h and it continued until 24 h. Moreover, the DiR fluorescence signal of the iRGD-LP-CUR-PIP-DiR group was stronger than that of the LP-CUR-DiR and LP-CUR-PIP-DiR groups. As shown in Figure 5C, the in vitro fluorescence signal of the heart, liver, spleen, lung, kidney and tumour in each group showed that iRGD increased the accumulation of drug in the liver, lung and tumour. The strongest fluorescence intensity was observed in the tumour and lung sites, verifying the tumour-targeting property of iRGD [29].

### 2.6. In Vivo Antitumour Activity of iRGD-LP-CUR-PIP

**Figure 6:**
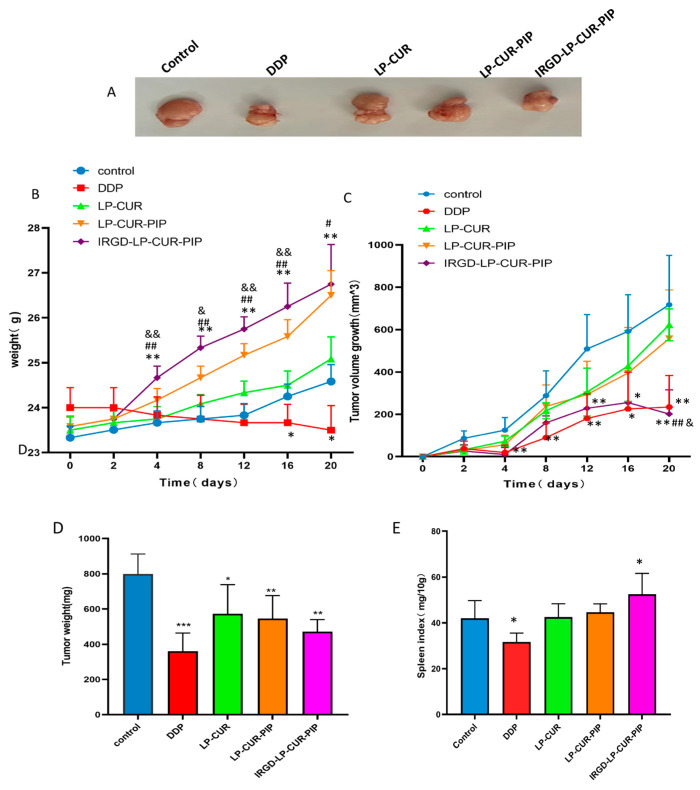
Antitumour activity of iRGD-LP-CUR-PIP in tumour-bearing mouse model. Notes: (**A**) Intraperitoneal injection of saline cisplatin (DDP), LP-CUR, LP-CUR-PIP and iRGD-LP-CUR-PIP into tumour-bearing mice. Images of isolated tumours after medical treatment. (**B**) Changes in body weight of nude mice in each group during the treatment period. ** *p* < 0.01 versus control, ^#^ *p* < 0.05 and ^##^ *p* < 0.01 versus LP-CUR and ^&^ *p* < 0.05 and ^&&^ *p* < 0.01 versus LP-CUR-PIP. (**C**) Changes in tumour volume in each group of tumour-bearing nude mice during the treatment period. * *p* < 0.05 and ** *p* < 0.01 versus control, ^##^ *p* < 0.01 versus LP-CUR and ^&^ *p* < 0.05 versus LP-CUR-PIP. (**D**) Tumour weight of tumour-bearing mice after treatment. * *p* < 0.05 and ** *p* < 0.01 and *** *p* < 0.001 versus control. (**E**) Spleen index of tumour-bearing mouse after treatment. * *p* < 0.05 versus control.

The following results were obtained after treating different groups of tumour-bearing nude mice (Figure 6). The iRGD-LP-CUR-PIP group achieved greater tumour changes by expending nude mice and isolating tumour combined with the control group from day 4, better than the LP-CUR and LP-CUR-PIP groups at day 20 (Figure 6A,C). iRGD-LP-CUR-PIP increased body weight significantly, whereas DDP decreased it (Figure 6B). On the 20th day after drug treatment, tumour-bearing mice were dissected and weighed to calculate tumour body mass and tumour inhibition rate. Compared with the control group, the tumour inhibition rates of LP-CUR, LP-CUR-PIP, iRGD-LP-CUR-PIP and DDP groups were 28.31%, 31.71%, 41.04% and 54.83%. After treatment, the spleen index of the mice was increased by iRGD-LP-CUR-PIP and decreased by DDP (Figure 6E). The antitumor efficacy results in subcutaneous tumour-bearing mice indicate that iRGD-LP-CUR-PIP treatment has stronger tumour growth inhibition than LP-CUR or LP-CUR-PIP treatment alone. Therefore, the combination of iRGD enhanced its accumulation in the tumour and improved its active targeting and therapeutic efficacy.

The results were attributed to the specific binding ability of iRGD to integrin receptors αvβ3 and αvβ5 overexpressed in tumour cells [30,31]. Upon cleavage by proteases, the C-terminal rule (C and R) motif is exposed and consequently binds to the neurexin-1 (NRP-1) receptor; the cleaved form of iRGD binds to NRP-1 and subsequently triggers NRP-1-dependent endocytosis, thereby enhancing tumour penetration [32]. iRGD can improve pharmacokinetics by ameliorating the problems of poor drug penetration and side effects [33]. In the present study, iRGD co-delivery was found to be an attractive strategy for selective enhancement of nanoparticle entry into tumours.

Studies have found that natural drug ingredients are natural immune modulators that play an antitumour role through immune regulation, thus highlighting the important role of the immune system in antitumour defence. The spleen is a major immune organ that can reflect the immune function of an organism directly. An increase in spleen index and the activation of immune activity contribute to antitumour properties [34]. Recent studies have found that many natural drug ingredients are immune modulators that play an antitumour role through immune regulation [35,36,37]. CUR and PIP are both plant chemicals with antioxidant and immune regulatory functions [38]. In this study, iRGD-LP-CUR-PIP showed high antitumor efficacy, mainly manifested as a significant reduction in tumour volume and an increase in body weight and spleen index, thereby improving immune regulatory function. This study provides a targeted treatment system based on natural ingredients to improve the antitumor efficacy of lung cancer, aiming to provide reference value for the use of iRGD peptide-modified co-loaded curcumin piperine liposomes in the medication and treatment of lung cancer.

## 3. Materials and Methods

### 3.1. Materials

CUR and PIP were purchased from Shanghai Maclin Biochemical Technology Co., Ltd. (Shanghai, China); Lecithin and cholesterol were purchased from Shanghai Genleaf Biotechnology Co., Ltd. (Shanghai, China); Phosphate buffer (pH 6.5) was purchased from Beijing Leigen Biotechnology Co., Ltd. (Beijing, China); iRGD-PEG-DSPE and mPEG-DSPE were provided by Guangzhou Carbowater Technology Co., Ltd. (Guangzhou, China); DIR iodide was purchased from Beijing Fuencyclopedia Biotechnology Co., Ltd. (Beijing, China); FBS, 1640 medium and 0.25% trypsin EDTA were purchased from Gibco.

The A549 cell line was obtained from the Stem Cell Bank, Chinese Academy of Sciences (Shanghai, China). The cells were cultured at 37 °C and 5% CO_2_ in 1640 complete medium (10% foetal bovine serum and 1% glutamine penicillin–streptomycin) and the culture medium was changed every other day and passaged at a 1:2 ratio every 2 days. Male Bagg Albino (BALB)/c nude mice weighing 20–23 g (5–6 weeks old) were provided by the Experimental Animal Center of Fujian University of Traditional Chinese Medicine (Fuzhou, China). They were housed and administered in a strictly sterile environment (the relative humidity was stabilised between 45% and 55% and the housing temperature was maintained at 23 °C–25 °C). All husbandry and handling of animals was conducted in strict accordance with the requirements of the university’s Laboratory Animal Care Facilities Authority.

### 3.2. Method

#### 3.2.1. Preparation of iRGD-LP-CUR-PIP

iRGD-LP-CUR-PIP was prepared via thin-film hydration. In brief, 10 mg CUR, 0.5 mg PIP, 516 mg lecithin, 100 mg cholesterol, 10 mg DSPE-PEG2000-iRGD and 40 mg DSPE-PEG2000 were dissolved in chloroform. The solvent was evaporated using an R-1001VN rotary evaporator (Zhengzhou Changchengke Industry and Trade Limited Company, Zhengzhou, China) via solid-film formation at 45 °C in a circular base flask. Then, 20 mL PBS (pH 6.5) was added to the flask and rotated in a rotary evaporator for 30 min at 60 °C to dissolve and fall off the membrane. Then, iRGD-LP-CUR-PIP was obtained via magnetic stirring at 60 °C for 20 min and ultrasonic probing at 400 W for 15 min.

#### 3.2.2. Characterisation of iRGD-LP-CUR-PIP

Morphology was observed using a transmission electron microscope. iRGD was added dropwise to 300-mesh copper mesh. After adsorption, it was stained with 2% phosphotungstic acid solution, dried at room temperature and observed. The particle size, polydispersity index (PDI) and zeta potential were measured using a nanoparticle size and potential analyser.

The encapsulation efficiency of liposomes was determined as described below. Ultrafiltered liposomes were demulsified in methanol and the supernatant was centrifuged at 13,000 R/min for 10 min and filtered through a 0.22 µm filter membrane. The chromatographic conditions were as follows: UPLC C18 column (2.1 mm × 100 mm, 1.7 μm), acetonitrile–4% glacial acetic acid aqueous solution (54:46, *v*/*v*) as mobile phase, flow rate of 0.2 mL/min, column temperature of 30 °C, detection wavelength of 366 nm and injection volume of 2 μL. The same amount of filtered liposomes was treated as described above.

(1) $$\mathrm{Encapsulation}\;\mathrm{efficiency}=\frac{CUR/PIP\;\mathrm{concentration}\;\mathrm{in}\;\mathrm{the}\;\mathrm{filtered}\;\mathrm{liposomes}}{CUR/PIP\;\mathrm{concentration}\;\mathrm{in}\;\mathrm{the}\;\mathrm{unfiltered}\;\mathrm{liposomes}}\times 100$$

#### 3.2.3. Stability of iRGD-LP-CUR-PIP

The release characteristics of iRGD-LP-CUR-PIP in vitro were investigated via membrane dialysis. An iRGD-LP-CUR-PIP solution (2.0 mL) was placed in a dialysis bag (mw 14000), which was then added to 40 mL PBS (pH levels 7.4 and 5.6) buffer containing 1% (*w*/*v*) Tween 80 and shaken on a shaking table at 140 R/min and 37 °C. Subsequently, 1.0 mL dialysate was withdrawn at 0.5, 1, 2, 4, 8, 24, 32, 48, 72, 96 and 144 h, separately, and the release medium was supplemented at the same temperature and volume. UPLC was used to determine the level of CUR in the dialysate and calculate the cumulative release rate of liposomes.

#### 3.2.4. In Vitro Cellular Uptake

A549 cells at the logarithmic growth phase were collected, counted and inoculated at 1 × 10^5^/well in six-well plates. They were incubated overnight at 37 °C with 5% CO_2_ to adhere to the wall. The culture medium was changed the next day, and LP-CUR, LP-CUR-PIP or iRGD-LP-CUR-PIP was diluted into 20 μg/mL of medium and incubated for 24 h under the same conditions as described above. The fluorescence intensity inside the cells was measured via stream cytometry to observe the uptake of different preparations.

The cells were incubated for 1 h at 4 °C or 37 °C after inoculation to explore the influence of temperature requirements on cell ingestion. Then, 20 μg/mL of iRGD-LP was added, and the cells were cultured for 1 h. Flow cytometry was subsequently used to detect cell fluorescence intensity.

The cells were inoculated in six-well plates after adding sucrose (137 mg/mL), a lattice protein-mediated endocytosis inhibitor; indomethacin (1 mg/mL), a non-steroidal anti-inflammatory; quercetin (80 μg/mL), a non-lattice protein-mediated endocytosis inhibitor; or amiloride (5.33 μg/mL), a macrocytic drinking inhibitor, separately, to explore the endocytosis mechanism. After the cells were incubated at 37 °C for 1 h, 20 μg/mL iRGD-LP was added, and the cells were incubated for 1 h. Then, cell fluorescence intensity was detected via flow cytometry.

#### 3.2.5. In Vitro Cell Invasion Inhibition

The influence of liposomes on A549 cell invasion was detected using the Transwell method. A549 cells were inoculated on the matrix glue of the upper chamber, and PBS, LP-CUR, LP-CUR-PIP and iRGD-LP-CUR-PIP were separately spiked in each upper chamber. Then, 1640 medium with 15% foetal bovine serum was spiked in the bottom chamber. The cells were stained with 4% paraformaldehyde at room temperature and 0.05% crystal violet solution for 20 min and observed under the microscope [39].

#### 3.2.6. In Vitro Cytotoxicity

The MTT method was used to determine in vitro cytotoxicity. In brief, A549 cells at the growth log phase were injected at a concentration of 1 × 10^4^/well into 96-well plates and incubated for 24 h under constant conditions. Then, iRGD-LP, LP-CUR, LP-CUR-PIP or iRGD-LP-CUR-PIP was diluted to a concentration of 0, 1.25, 2.5, 5, 5, 10, 20, 40 or 80 μg/mL medium and further incubated at 37 °C for 24, 48 or 72 h. Next, 20 μL MTT (5 mg/mL) was added to each well, which was then incubated for 4 h. The supernatant was discarded and 150 μL of dimethyl sulfoxide was added to each well and shaken for 10 min. Finally, the absorbance (A) value at 490 nm was measured using Multiskan Sky enzyme standard. Survival was calculated using the method in which cells cultured with medium alone were considered as 100% viable controls [40].

#### 3.2.7. Imaging of Biodistribution In Vivo

An A549 tumour-bearing nude mouse model was prepared by subcutaneously injecting 0.2 mL A549 cell (5 × 10^6^) suspension into the right axilla (after routine sterilisation) of male BALB/c nude mice. When the subcutaneous tumour mass reached 150–200 mm^3^, the nude mice were injected in the tail vein (after routine sterilisation) with 200 μL saline, LP-CUR-PIP-DiR or iRGD-LP-CUR-PIP-DiR at a dose of 50 mg/kg. Afterwards, anaesthesia was applied using isoflurane (1.5%) and after 10 min, the mice were placed in a small animal live imager and scanned by IVIS spectroscopy (Perkin Elmer) at an absorption wavelength of 748 nm and an emission wavelength of 780 nm with a filter mounted at 710 ex/760 em. Bioluminescence imaging was performed at 2, 4, 8, 12 and 24 h after drug administration. The system was set to an exposure time of 60 s per image. Living Image 4.4 software was used to analyse the fluorescence signal intensity in tumour-bearing nude mice.

#### 3.2.8. In Vivo Antitumour Activity of iRGD-LP-CUR-PIP

An A549 tumour-bearing nude mouse model was successfully prepared in accordance with the above method. When the volume of tumour reached 150–200 mm^3^, the BALB/c mice were randomly divided into five groups (each group had six animals): control group, where saline was injected intraperitoneally (IP); LP-CUR group (50 mg/kg, IP, qd for 20 days); LP-CUR-PIP group (50 mg/kg, IP, qd); iRGD-LP-CUR-PIP group (50 mg/kg, IP, qd); and DDP group (2 mg/kg, IP, qd). During the whole experiment, the survival of mice was observed every day, and their body weight was recorded every 2 days, and the length and diameter of the tumours were measured with a Vernier calliper. The tumour volume was calculated as follows: V = length (mm) × width (mm^2^) × 0.5. Antitumor rate% = (1- average tumour mass of the experimental groups/average tumour mass of the control groups) × 100%.

#### 3.2.9. Statistical Analysis

SPSS version 18.0 was used for data analysis. All data were represented as mean ± standard deviation. One-way ANOVA was used for comparison between groups and the Bonferroni-corrected postoperative test was used for final comparison between groups. *p* < 0.05 was considered statistically significant.

## 4. Conclusions

In this study, a novel iRGD-LP-CUR-PIP was prepared to evaluate its antitumour activity against A549 lung cancer cells in vitro and in vivo. As verified by in vitro cellular assays, iRGD-LP-CUR-PIP significantly inhibited the invasion of A549 cells and the tumour-targeting and -penetrating activities of iRGD-modified LP were confirmed via in vivo biodistribution analysis. The anti-lung tumour activity of iRGD-LP-CUR-PIP was confirmed in A549 tumour-bearing mice. iRGD-LP-CUR-PIP is a peptide-modified liposome with significant antitumour activity for tumour targeting and penetration.

## Figures and Tables

**Table 1:** The characteristics of iRGD-LP-CUR-PIP (*n* = 3).

	Average Particle Size (nm)	Polydispersity	Zeta Potential (mV)	Entrapment Efficiency (%)	
				CUR	PIP
LP-CUR mean ± SD	86.0 ± 2.5	0.341 ± 0.009	−0.4 ± 0.1	94.26% ± 1.82	-
LP-CUR-PIP mean ± SD	95.5 ± 1.8	0.337 ± 0.021	−0.3 ± 0.1	90.30% ± 0.62	92.46% ± 0.76
iRGD-LP-CUR-PIP mean ± SD	93.9 ± 0.9	0.200 ± 0.015	0.3 ± 0.1	95.23% ± 1.08	97.04% ± 1.16

## Data Availability

The datasets used and analysed during the current study are available from the corresponding author upon reasonable request.

## Sample Availability

Not available.
